# Protective host defense against disseminated candidiasis is impaired in mice expressing human interleukin-37

**DOI:** 10.3389/fmicb.2014.00762

**Published:** 2015-01-07

**Authors:** Frank L. van de Veerdonk, Mark S. Gresnigt, Marije Oosting, Jos W. M. van der Meer, Leo A. B. Joosten, Mihai G. Netea, Charles A. Dinarello

**Affiliations:** ^1^Department of Medicine, University of Colorado DenverDenver, CO, USA; ^2^Department of Medicine, Radboud University Nijmegen Medical CenterNijmegen, Netherlands; ^3^Radboud Center for InfectionNijmegen, Netherlands

**Keywords:** IL-37, *Candida*, TNFα, inflammation, neutrophils, antifungal host defense, IL-1F7

## Abstract

The effect of the anti-inflammatory cytokine interleukin-37 (IL-37) on host defense against *Candida* infections remains unknown. We assessed the role of IL-37 in a murine model of disseminated candidiasis using mice transgenic for human IL-37 (hIL-37Tg). Upon exposure to *Candida albicans* pseudohyphae, macrophages from hIL-37Tg mice release 39% less TNFα compared to cells from wild-type (WT) mice (*p* = 0.01). *In vivo*, hIL-37Tg mice displayed a decreased capacity to recruit neutrophils to the site of infection. These defects were associated with increased mortality and organ fungal growth in hIL-37Tg compared to WT mice. We conclude that IL-37 interferes with the innate protective anti-*Candida* host response by reducing the production of proinflammatory cytokines and suppressing neutrophil recruitment in response to *Candida,* resulting in an increased susceptibility to disseminated candidiasis.

## INTRODUCTION

The interleukin-1 (IL-1) family includes inflammatory cytokines (such as IL-1α, IL-1β, IL-18, and IL-33) that activate target cells through a receptor-mediated mechanism; anti-inflammatory cytokine antagonists (such as IL-1Ra) block IL-1-dependent activation by competing for binding to the IL-1 receptors (Dinarello, 2011). Twelve years ago, using *in silico* searches, additional members of the IL-1 family namely IL-36, IL-37, and IL-38 were discovered, but only recently have their properties been studied (Dinarello, 2013). In the case of IL-37, this cytokine has emerged as fundamental inhibitor of innate inflammation using mice transgenic for human IL-37 as well as reducing endogenous IL-37 in human blood monocytes (Nold et al., 2010). For example, mice transgenic for human IL-37 are protected against systemic endotoxemia, chemical induced colitis, ConA hepatitis, and ischemic reperfusion injury, as reviewed in Dinarello and Bufler (2013). The mechanism by which IL-37 affords anti-inflammatory protection can be via a caspase-1-dependent nuclear translocation or by an extracellular mechanism engaging the IL-18 receptor (Bulau et al., 2014). It is important however, to recognize that anti-inflammatory strategies based upon IL-37 could have a negative impact on susceptibility to infections by inhibiting host defense. Since in models of live infection blocking inflammation mediated by cytokines such as IL-1 or TNFα can be either detrimental or beneficial for the outcome of the host, we therefore carried-out studies of disseminated candidiasis in mice expressing human IL-37.

## MATERIALS AND METHODS

### ANIMALS

Transgenic mice expressing human IL-37 (hIL-37Tg) have been previously described Nold et al. (2010). Control C57BL/6J (WT) mice were purchased from the Jackson Laboratory and maintained under specific-pathogen free conditions. The mice were fed sterilized laboratory chow (Hope Farms, Woerden, The Netherlands) and water *ad libitum*. The experiments were approved by the Ethics Committee on Animal Experiments of Radboud University Nijmegen.

### *Candida albicans* AND GROWTH CONDITIONS

*Candida albicans* ATCC MYA-3573 (UC 820), a strain well described elsewhere (Lehrer and Cline, 1969), was used in all experiments. *Candida* was grown overnight in Sabouraud broth at 37∘C, cells were harvested by centrifugation, washed twice, and resuspended in culture medium (RPMI-1640 Dutch modification, ICN Biomedicals, Aurora, OH, USA; van der Graaf et al., 2005). To generate pseudohyphae, *C. albicans* blastoconidia were grown at 37∘C in culture medium, adjusted to pH 6.4 by using hydrochloric acid. Pseudohyphae were killed at 100∘C for 1 h and resuspended in culture medium to a hyphal inoculum size that originated from 10^6^ blastoconidia per ml (referred to as 10^6^ pseudohyphae per ml).

### CYTOKINE RESPONSES OF *C. albicans*-STIMULATED MACROPHAGES AND SPLENOCYTES

Resident peritoneal macrophages were harvested from groups of five hIL-37Tg and wild-type (WT) mice by injecting 4 ml of sterile phosphate-buffered saline (PBS) intrapertitoneal containing 0.38% sodium citrate (Kullberg et al., 1993). After washing, the cells were resuspended in culture medium in 96-well microtiter plates (Greiner, Alphen, The Netherlands) at 10^5^ cells/well, in a final volume of 200 μl. The cells were stimulated with either control medium or heat killed *C. albicans* at 1 × 10^7^ microorganisms/ml. After 24 h of incubation at 37∘C, the plates were centrifuged (500 g, 10 min), and the supernatants were removed. The cells were lysed with three freeze-thaw cycles. Samples were stored at -80∘C until cytokine assays were performed.

To assess cytokine production in splenocytes, spleen were gently squeezed into a sterile 200 mm filter chamber. The cells were washed and resuspended in RPMI 1640, counted in a Bürker chamber and adjusted to 5 × 10^6^/ml. 200 μL of the cell suspension was stimulated with 1 × 10^7^ heat-killed *C. albicans*/ml. Measurement of TNFα and IL-6 concentrations was performed in supernatants collected after 48 h of incubation at 37∘C in 5% CO_2_ in 48-well plate.

### CYTOKINE ASSAYS

TNFα was determined by specific radioimmunoassay (detection limit 20 pg/ml), as previously described Netea et al. (1996). IL-6 concentrations were determined by a commercial ELISA (Biosource, Camarillo, CA, USA, detection limit 16 pg/ml) according to the instructions of the manufacturer.

### *C. albicans* INFECTION MODEL

hIL-37Tg mice and WT mice were injected intravenously with *C. albicans* blastoconidia (1 × 10^6^ CFU/mouse for survival and 5 × 10^5^ CFU/mouse for testing fungal burden) in a 100 μl volume of sterile pyrogen-free PBS. Survival was assessed daily for 2 weeks. Subgroups of five animals were killed on days 3 and 7 of infection. To assess the tissue outgrowth of the microorganisms, the kidneys of the sacrificed animals were removed aseptically, weighed, and homogenized in sterile saline in a tissue grinder. The number of viable *Candida* cells in the tissues was determined by plating serial dilutions on Sabouraud dextrose agar plates. The CFU were counted after 24 h of incubation at 37∘C, and expressed as log CFU/g tissue.

### *Candida* OUTGROWTH IN PMN DEPLETED MICE

Sexually mature female C57bl/6 mice (8–12 weeks old, weights 20–25 g) were used. The animals were kept under routine laboratory conditions (21–22∘C, relative humidity 60% and a 12 h light–dark cycle), fed a standard commercial pellet diet (RHM, Hope Farms, The Netherlands), and given acidified tap water *ad libitum*. The mice were injected intraperitoneal with rat anti-mouse GR1 or isotype control (100 μg in 200 μl PBS; eBioscience) 4 days prior to the experiment to deplete the mice of PMNs. On day zero, the mice were injected intraperitoneal with 1 × 10e5 live *C. albicans* conidia. After 2 h the mice were anesthetized with isoflurane and blood samples were obtained for measurement of blood cells counts. Subsequently, the mice were sacrificed by cervical dislocation. The number of viable *Candida* cells in the peritoneal lavage was determined by plating serial dilutions on Sabouraud dextrose agar plates and the CFU were counted after 24 h of incubation at 37∘C and expressed as log CFU.

### *IN VITRO* CYTOKINE PRODUCTION BY PRIMED SPLENOCYTES

To assess cytokine production during infection, primed spleen cells from mice on day 7 of infection with 2 × 10^5^ CFU of *C. albicans* per mouse were stimulated *in vitro* with heat-killed *Candida* conidia or pseudohyphae (1 × 10^6^ microorganisms/ml). Spleen cells were washed and resuspended in RPMI1640, counted in a Bürker chamber and the number was adjusted to 5 × 10^6^/ml. 200 μL of the cell suspension was stimulated with 1 × 10^6^ heat killed *C. albicans*/ml. Measurement of IFNγ, IL-10, and IL-17 concentrations was performed in supernatants collected after 48 h of incubation at 37∘C in 5% CO_2_ in 48-well plate.

### PHAGOCYTOSIS AND KILLING OF *C. albicans* BY MACROPHAGES

Phagocytosis and killing of *C. albicans* blastoconidia were assessed as described elsewhere (Vonk et al., 2006). Exudate peritoneal phagocytes from groups of five hIL-37Tg mice and WT mice were elicited by an i.p. injection of 10% proteose peptone. After 72 h, cells were collected in separate sterile tubes by washing the peritoneal cavity with 4 ml of ice-cold PBS that contained 50 U/ml heparin. Phagocytes were centrifuged (for 10 min at 2250 g), counted in a Bürker chamber, and resuspended in culture medium. 5 × 10^5^ cells were dispensed into the wells of a 96-well flat bottom plate (Costar), allowed to adhere for 2 h, and washed to remove non-adherent cells. Subsequently, the cells were incubated with 1 × 10^4^ CFU *C. albicans*, which were opsonized for 45 min at 24∘C in modified Eagle’s medium (MEM; Gibco Life Technologies) containing 2.5% fresh mouse serum (effector:target ratio, 40:1). After 15 min, supernatants were aspirated, and monolayers were gently washed with MEM to remove non-ingested microorganisms. The supernatant and well washings that contained the non-ingested *Candida* blastoconidia were plated in serial dilutions on Sabouraud agar plates. The percentage of phagocytosed microorganisms was defined as [1–(number of uningested CFU/CFU at the start of incubation)] × 100. Killing of *C. albicans* by phagocytes was assessed in the same monolayers. After removal of the non-phagocytized *Candida* blastoconidia, 200μl of culture medium, consisting of Sabouraud in MEM (50% vol/vol), was added to the monolayers. After 3 h of incubation at 37∘C in air and 5% CO_2_, the wells were scraped gently with a plastic paddle and washed with 200μl distilled H_2_O to achieve lysis of phagocytes in three cycles, and 10-fold dilutions of each sample were spread on Sabouraud agar plates and incubated at 37∘C for 24 h. The percentage of yeast killed by the phagocytes was determined as follows: [1–(CFU after incubation/number of phagocytized CFU)] (Nold et al., 2010) × 100. Phagocyte-free incubations of blastoconidia were included as a control for yeast viability. In earlier experiments we have shown that 90–97% of attached/internalized *C. albicans* are intracellular (Vonk et al., 2006).

### STATISTICAL ANALYSIS

The differences between groups were analyzed by the Mann–Whitney *U*-test. The level of significance between groups was set at *p* < 0.05. The data are presented as cumulative results of all experiments performed.

## RESULTS

### hIL-37Tg MICE ARE MORE SUSCEPTIBLE TO DISSEMINATED *C. albicans* INFECTION

**FIGURE 1:**
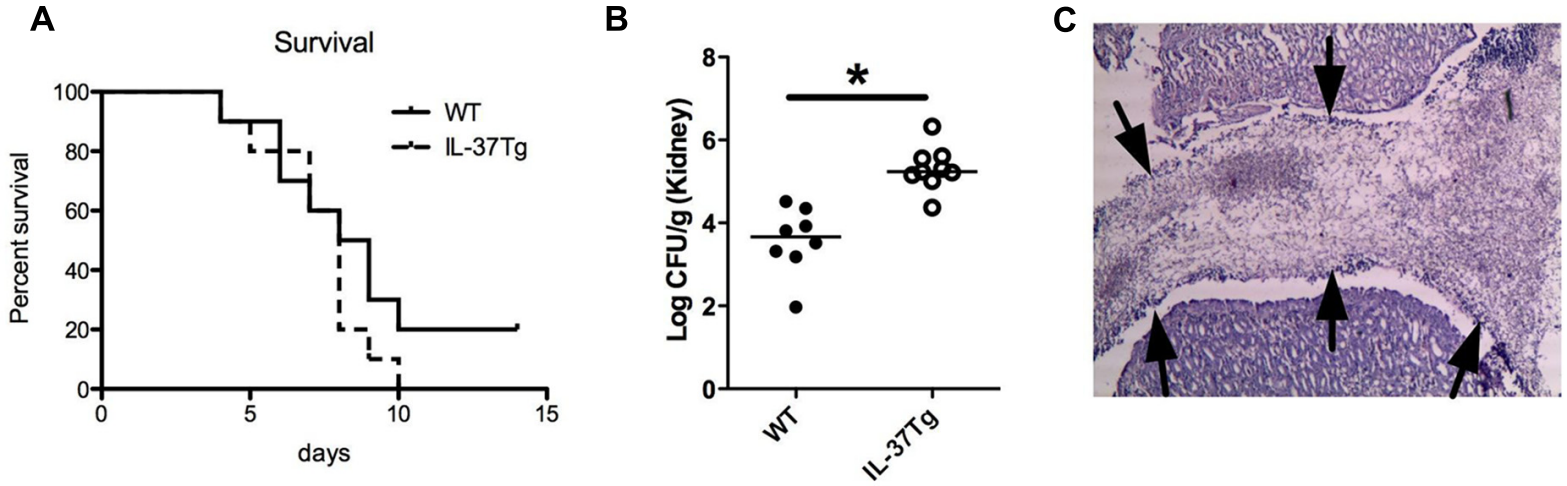
**hIL-37Tg mice are highly susceptible to disseminated *C. albicans* infection. (A)** Survival of hIL-37Tg mice and WT mice intravenously infected with live *C. albicans* yeast (1 × 10e6 CFU). **(B)** hIL-37Tg mice and WT mice were infected i.v. with *C. albicans* blastoconidia (5 × 10e5 CFU/mouse). Subgroups of animals (a total of nine mice/group/time point, in two separate experiments) were killed on day 7 of infection, and fungal outgrowth was assessed in the kidneys. **(C)** Histology representing the accumulation of *C. albicans* hyphae (fungal ball) in the pyelum of hIL-37Tg mice. Arrows point to the edge of the fungal ball present in the pyelum of the kidney **(A)**. Survival was assessed daily for 2 weeks. (*n* = 10/group; **B**) Mann–Whitney *U*-test, *p< 0.05.

To investigate the role of IL-37 in the host defense against invasive *C. albicans* infection, hIL-37Tg mice, and WT mice were infected with *C. albicans* blastospores. The survival during disseminated candidiasis was 20% for WT-type mice after 2 weeks, whereas all mice had died by day 10 in the hIL-37Tg group (**Figure 1A**). The fungal burden in the kidneys, the target organ of disseminated candidiasis in mice (Spellberg et al., 2005), was not significantly different at day 3 (mean ± SD; WT: 3.6 ± 0.83, IL-37Tg 3.8 ± 0.44 log CFU/g kidney), and 1-log higher in the hIL-37Tg mice than in WT mice on day 7 of infection (*p* < 0.05; **Figure 1B**). Histology revealed an accumulation of *Candida* in the pyelum of the kidneys of hIL-37Tg mice which was not observed in the WT mice (**Figure 1C**).

### NEUTROPHIL RECRUITMENT AND PHAGOCYTOSIS AND KILLING OF *C. albicans*

**FIGURE 2:**
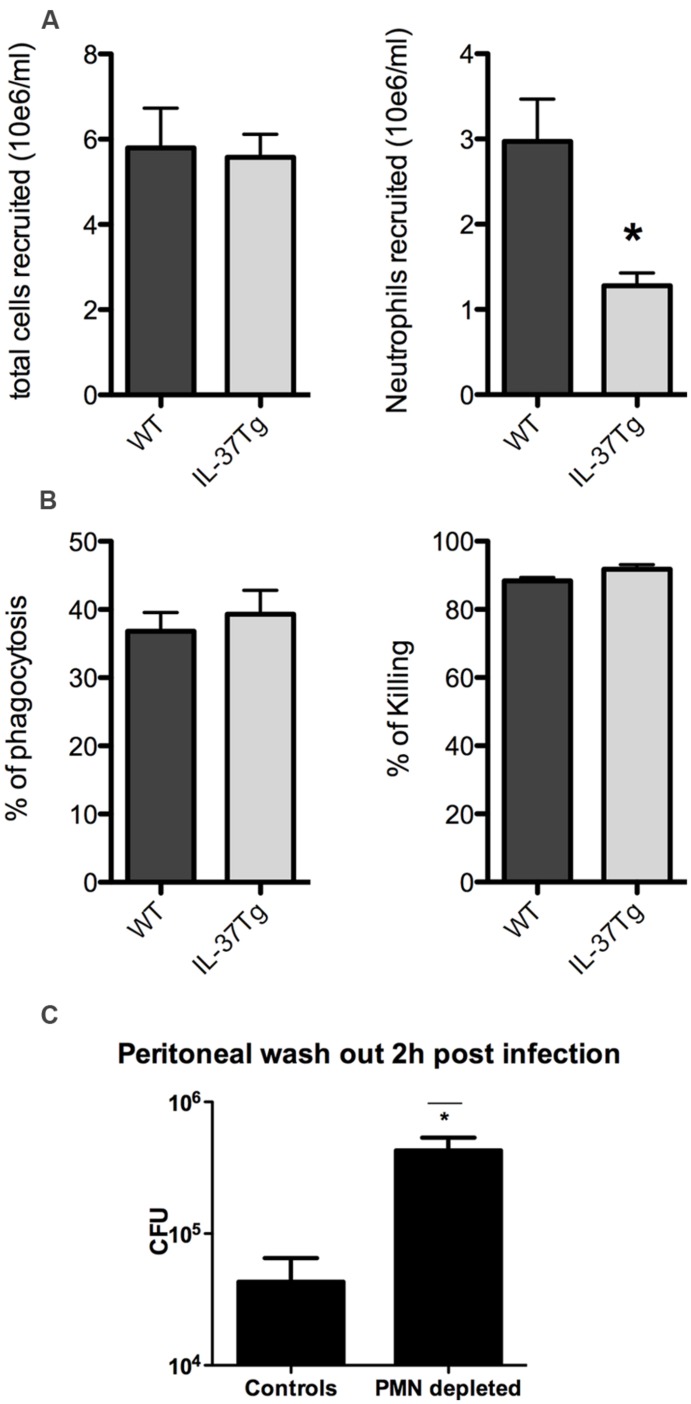
**Neutrophil recruitment and phagocytosis and killing of *C. albicans.* (A)** Absolute numbers of total cells and absolute numbers of neutrophils recruited to the peritoneal cavity of WT and hIL-37Tg mice 4 h after intraperitoneal injection of 1 × 10e7 heat killed *C. albicans* yeasts. **(B)** Percentage of *Candida* blastoconidia of the initial inoculum that was ingested by macrophages of hIL-37Tg and WT mice after 15 min of phagocytosis and percentage of phagocytized *Candida* blastoconidia that was killed after incubation at 37∘C for 3 h is shown. The results are pooled data with a total of five hIL-37Tg mice and five WT mice (mean ± SEM, *p < 0.05, Students *t*-test). **(C)** Fungal outgrowth of peritoneal wash-outs in neutrophil (PMN) depleted and non-PMN depleted WT mice 2 h after intraperitoneal infection with live *Candida*. The results are pooled data with a total of four hIL-37Tg mice and four WT mice (mean ± SEM, *p < 0.05, Students *t*-test).

In order to understand the enhanced lethality and greater fungal burden of the hIL-37Tg mice, we investigated the dynamics of the influx and function of neutrophils. Peritoneal cells were harvested and counted 4 h after intraperitoneal injection of heat-killed *Candida*. A significant lower influx of neutrophils was apparent 4 h after challenge with heat killed *Candida* in the hIL37Tg mice compared to WT mice (**Figure 2A**). Phagocytosis of *C. albicans* by hIL-37Tg cells was similar to that by cells of WT mice (37 vs. 39% phagocytized in 15 min; *p* > 0.05). hIL-37Tg cells killed 92% of phagocytized *Candida* blastoconidia after 3 h, which was not different from the killing activity of cells from WT cells (88%, *p* > 0.05, **Figure 2B**).

To assess whether the decrease of neutrophils within the first hours has a significant biological effect, we investigated the difference of fungal burden between neutrophil depleted mice and non-neutrophil depleted mice in a short *in vivo Candida* infection model. The lack of neutrophils at the site of infection resulted in a log increase in fungal growth within 2 h compared to mice in which neutrophil influx was normal (**Figure 2C**). These differences support the importance of early influx of neutrophils in restricting *Candida* growth *in vivo*.

### IL-37 INHIBITS PROINFLAMMATORY CYTOKINE PRODUCTION BY MACROPHAGES AND SPLENOCYTES STIMULATED WITH *C. albicans*

**FIGURE 3:**
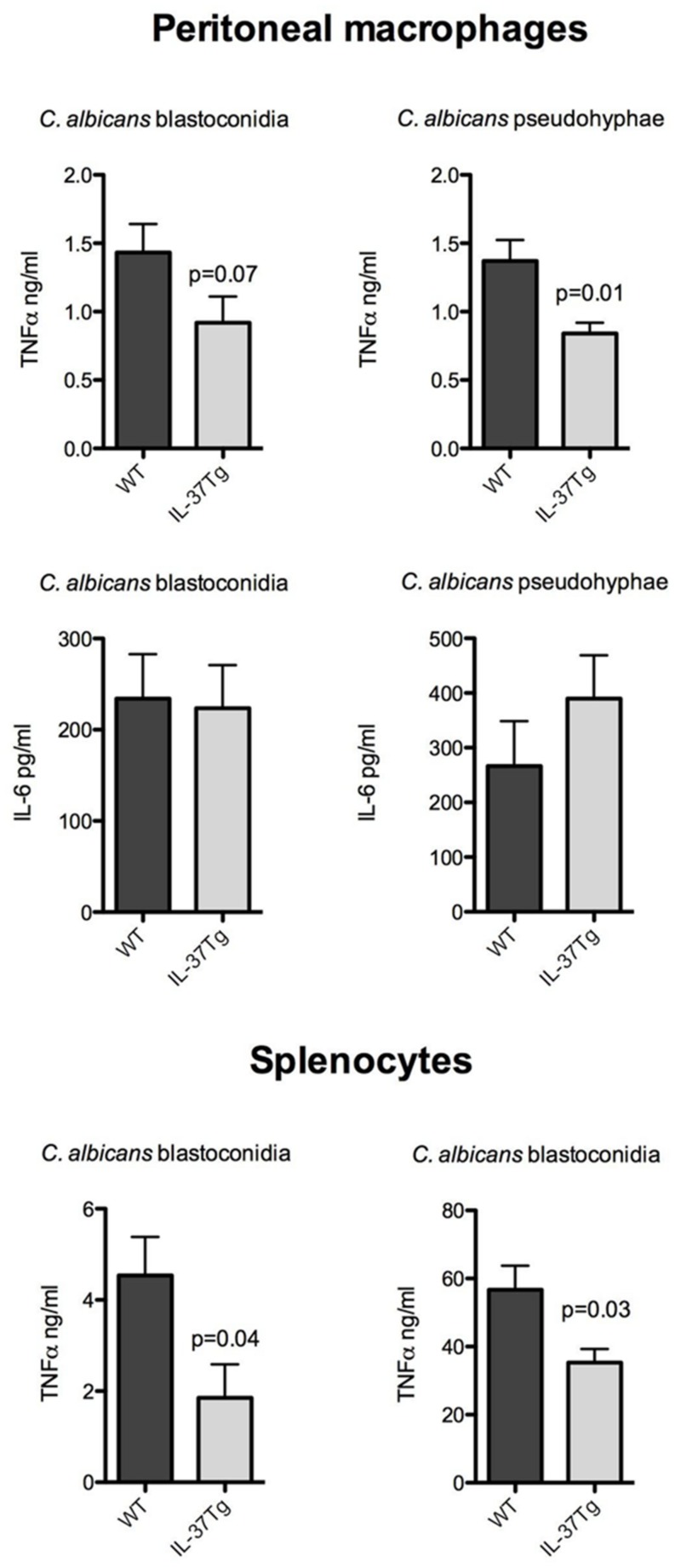
**IL-37 inhibits proinflammatory cytokine production by macrophages and splenocytes stimulated with *C. albicans*. (A)** Resident peritoneal macrophages from hIL-37Tg mice and WT mice were stimulated with either 1 × 10e7 microorganisms/ml heat-killed *Candida* blastoconidia or pseudohyphae. **(B)** Splenocytes from hIL-37Tg mice and WT mice were stimulated with either 1 × 10e7 microorganisms/ml *Candida* blastoconidia or pseudohyphae. Production of TNFα and IL-6 in the supernatants was measured after 24 h of stimulation at 37∘C for macrophages and 48 h of stimulation at 37∘C for splenocytes. The results are pooled data with a total of seven hIL-37Tg mice and six WT mice (mean ± SEM, *p < 0.05, Mann–Whitney *U*-test).

To investigate the inhibitory effect of IL-37 at the level of cytokine production, resident peritoneal macrophages, or naïve splenocytes of hIL-37Tg and WT mice were exposed to heat-killed *C. albicans* blastoconidia and pseudohyphae *in vitro*. Cytokine production by unstimulated macrophages of each mouse strain was below the detection limit for TNFα and IL-6. However, after stimulation with *C. albicans* pseudohyphae, the production of TNFα was significantly lower in macrophages from the hIL-37Tg mice compared to cells from WT mice. There were no differences in the production of IL-6 in the same cultures (**Figure 3**).

### hIL-37Tg MICE DISPLAY INCREASED Th17 AND IL-10 RESPONSES

**FIGURE 4:**
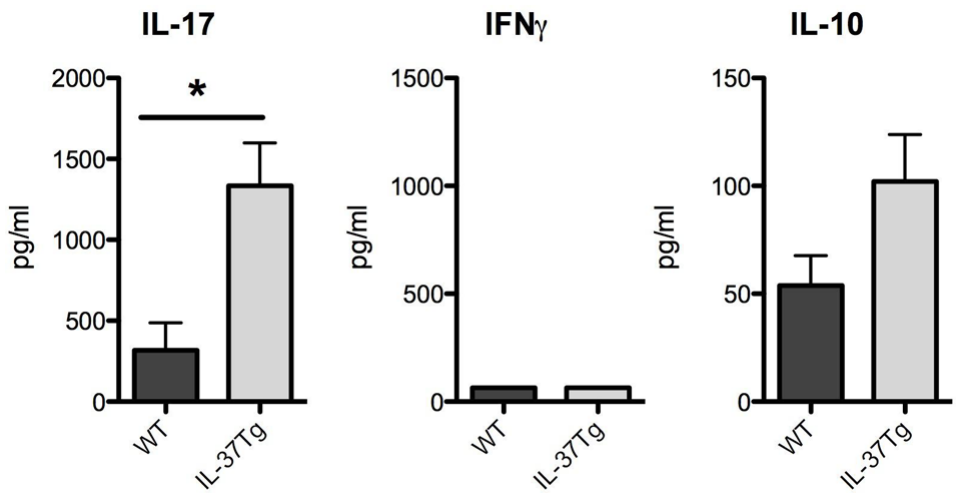
**hIL-37Tg mice display increased Th17 and IL-10 responses.** Splenocytes from WT and hIL-37Tg mice were re-stimulated with RPMI (gray bars) or heat-killed 1 × 10^6^ *C. albicans* pseudohyphae cells/ml (black bars) 7 days after intravenous infection with *C. albicans*. IL-17, IFNγ, and IL-10 were measured 48 h after stimulation with ELISA. *p < 0.05; *n* = 5 mice per group. Bars indicate mean ± SEM.

Proinflammatory T helper responses such as Th1 and Th17 responses play an important role in fungal host defense (Gow et al., 2012). The Th17 response during disseminated fungal infection can be both protective and detrimental (Huang et al., 2004; Zelante et al., 2007; Lin et al., 2009). To study the impact of IL-37 on proinflammatory adaptive immune responses, we investigated the Th1 and Th17 characteristic cytokines IFNγ and IL-17. Splenocytes isolated from hIL-37Tg mice with a higher fungal burden at day 7 produced significant more IL-17 in response to *C. albicans* pseudohyphae (**Figure 4**). IFNγ production in response to *C. albicans* pseudohyphae was undetectable in WT and hIL-37Tg splenocytes at day 7 (**Figure 4**). Since IL-37 mainly has anti-inflammatory effects (Nold et al., 2010; Boraschi et al., 2011), we investigated levels of IL-10 in cells from IL-37Tg There was no significant difference in IL-10 production in splenocytes from hIL-37Tg compared to WT cells in response to *C. albicans*, although hIL-37Tg mice showed a trend toward an increased IL-10 production (**Figure 4**).

## DISCUSSION

In the present study, we demonstrate that overexpression of IL-37 is detrimental for the early host defense against *C. albicans* in a murine model of disseminated candidiasis. This conclusion is based on the increased fungal load in the kidney, the target organ of disseminated candidiasis, in hIL-37Tg mice compared to WT mice. The greater outgrowth of the fungus in the tissues is most probably due to impaired early influx of neutrophils into the infected tissues, which in turn may be due to lower production of neutrophil-inducing cytokines, such as TNF. Therefore, the presence of IL-37 in the early stages of infection results in an impaired innate immune response that is essential to limit fungal dissemination.

Recently, several biological functions of the cytokine IL-37 have been described. IL-37 has a protective role in a murine model of lethal endotoxemia (Nold et al., 2010; Bulau et al., 2011), and it has been reported that mice overexpressing IL-37 are protected from DSS colitis (McNamee et al., 2011). These reports point to significant anti-inflammatory properties of IL-37. In the present study we extend these findings to a live infection model, the murine model of disseminated candidiasis, in which we assessed whether IL-37 hampers the innate immune defense against a fungal infection. We observed that mice overexpressing IL-37 were more susceptible to invasive candidiasis, due to the inability to control fungal growth in the kidney during infection. This effect is at least partly due to a defective neutrophil recruitment, a cell population central for the local antifungal host defense (Kullberg et al., 1990), and this supports the previously reported finding that leukocyte recruitment to the site of inflammation is decreased in hIL-37Tg mice (McNamee et al., 2011). The importance of neutrophils in the control of *Candida* outgrowth *in vivo* is further supported in this study by the observation that neutrophil depleted mice have a log increase within 2 h in fungal burden compared to mice that have a normal neutrophil influx. In addition, macrophages and splenocytes overexpressing IL-37 produced significantly less TNFα than WT macrophages when exposed to *C. albicans*. This is in line with other studies, showing that macrophages from hIL-37Tg mice produce less TNF and that IL-37 overexpression reduces TNF-dependent inflammation in a murine model of colitis (McNamee et al., 2011). An involvement of defective TNF production in the increased susceptibility of hIL-37Tg mice to disseminated candidiasis is also consistent with earlier studies showing that mice lacking TNF or the TNF receptor are highly susceptible to disseminated candidiasis (Netea et al., 1999).

Another observation of this study is that neutrophils from hIL-37Tg mice do not display an intrinsic defect in phagocytosis and killing, suggesting that IL-37 does not directly impair neutrophil function. The increased Th17 response in hIL-37Tg mice present on day 7 most likely reflects an increased cellular immune response due to higher fungal burden (antigen) exposure. Whether this increased Th17 response has directly contributed to the higher susceptibility of hIL-37Tg mice to disseminated candidiasis remains to be determined. Interestingly, hIL-37Tg splenocytes showed a consistent trend at day 7 toward an increased production of the anti-inflammatory cytokine IL-10 when exposed to *C. albicans*. This observation is consistent with the increased IL-10 production found in hIL-37Tg mice with experimental colitis, and supports the concept that IL-37 can contribute to systemic anti-inflammatory effects (McNamee et al., 2011).

In conclusion, IL-37 reduces proinflammatory cytokine production induced by *Candida* in macrophages, and decreases neutrophil recruitment *in vivo* in response to *C. albicans*. These data highlight the potent anti-inflammatory effects of IL-37, and underline that the timing of IL-37 expression in the tissue at the site of inflammation is critical for the outcome of the host during inflammatory processes.

## Conflict of Interest Statement

The authors declare that the research was conducted in the absence of any commercial or financial relationships that could be construed as a potential conflict of interest.

## References

[B1] Boraschi D. Lucchesi D. Hainzl S. Leitner M. Maier E. Mangelberger D. (2011). IL-37: a new anti-inflammatory cytokine of the IL-1 family. *Eur. Cytokine Netw.* 22 127–147 10.1684/ecn.2011.0288 22047735

[B2] Bulau A. M. Fink M. Maucksch C. Kappler R. Mayr D. Wagner K. (2011). In vivo expression of interleukin-37 reduces local and systemic inflammation in concanavalin a-induced hepatitis. *Sci. World J.* 11 2480–2490 10.1100/2011/968479 PMC3253525 22235179

[B3] Bulau A. M. Nold M. F. Li S. Nold-Petry C. A. Fink M. Mansell A. (2014). Role of caspase-1 in nuclear translocation of IL-37, release of the cytokine, and IL-37 inhibition of innate immune responses. *Proc. Natl. Acad. Sci. U.S.A.* 111 2650–2655 10.1073/pnas.1324140111 24481253 PMC3932872

[B4] Dinarello C.A. (2011). Interleukin-1 in the pathogenesis and treatment of inflammatory diseases. *Blood* 117 3720–3732 10.1182/blood-2010-07-273417 21304099 PMC3083294

[B5] Dinarello C.A. (2013). Overview of the interleukin-1 family of ligands and receptors. *Semin. Immunol.* 25 389–393 10.1016/j.smim.2013.10.001 24275600

[B6] Dinarello C.A. Bufler P. (2013). Interleukin-37. *Semin. Immunol.* 25 466–468 10.1016/j.smim.2013.10.004 24275599

[B7] Gow N.A van de Veerdonk F. L. Brown A. J. Netea M. G. (2012). *Candida albicans* morphogenesis and host defence: discriminating invasion from colonization. *Nat. Rev. Microbiol.* 10 112–122 10.1038/nrmicro2711 22158429 PMC3624162

[B8] Huang W. Na L. Fidel P.L. Schwarzenberger P. (2004). Requirement of interleukin-17A for systemic anti-*Candida albicans* host defense in mice. *J. Infect. Dis.* 190 624–631 10.1086/422329 15243941

[B9] Kullberg B.J. van’t Wout J.W. Hoogstraten C. Van Furth R. (1993). Recombinant interferon-γ enhances resistance to acute disseminated *Candida albicans* infection in mice. *J. Infect. Dis.* 168 436–443 10.1093/infdis/168.2.436 8335982

[B10] Kullberg B.J. van’t Wout J.W. Van Furth R. (1990). Role of granulocytes in enhanced host resistance to *Candida albicans* induced by recombinant interleukin-1. *Infect. Immun.* 58 3319–3324. 2144844 10.1128/iai.58.10.3319-3324.1990 PMC313656

[B11] Lehrer R.I. Cline M. J. (1969). Interactions of *Candida albicans* with human leukocytes and serum. *J. Bacteriol.* 98 996–1004. 4182532 10.1128/jb.98.3.996-1004.1969 PMC315286

[B12] Lin L. Ibrahim A. S. Xu X. Farber J. M. Avanesian V. Baquir B. (2009). Th1-Th17 cells mediate protective adaptive immunity against *Staphylococcus aureus* and *Candida albicans* infection in mice. *PLoS Pathog.* 5:e1000703 10.1371/journal.ppat.1000703 PMC2792038 20041174

[B13] McNamee E. N. Masterson J. C. Jedlicka P. McManus M. Grenz A. Collins C. B. (2011). Interleukin 37 expression protects mice from colitis. *Proc. Natl. Acad. Sci. U.S.A.* 108 16711–16716 10.1073/pnas.1111982108 21873195 PMC3189085

[B14] Netea M. G. Demacker P. N. M. Kullberg B. J. Boerman O. C. Verschueren I. Stalenhoef A. F. (1996). Low-density-lipoprotein receptor deficient mice are protected against lethal endotoxinemia and severe gram-negative infections. *J. Clin. Invest.* 97 1366–1372 10.1172/JCI118556 8617867 PMC507194

[B15] Netea M. G. Van Tits L. J. H. Curfs J. H. A. J. Amiot F. Meis J. F. G. M. van der Meer J. W. M. (1999). The increased susceptibility of TNFαLTα double knock-out mice to systemic candidiasis is due to defective recruitment and phagocytosis by neutrophils. *J. Immunol.* 163 1498–1505. 10415052

[B16] Nold M.F. Nold-Petry C. A. Zepp J. A. Palmer B. Bufler B. E. Dinarello C. A. (2010). IL-37 is a fundamental inhibitor of innate immunity. *Nat. Immunol.* 11 1014–1022 10.1038/ni.1944 20935647 PMC3537119

[B17] Spellberg B. Ibrahim A. S. Edwards J. E. Jr. Filler S. G. (2005). Mice with disseminated candidiasis die of progressive sepsis. *J. Infect. Dis.* 192 336–343 10.1086/430952 15962230

[B18] van der Graaf C.A. A. Netea M. G. Verschueren I. van der Meer J. W. M. Kullberg B. J. (2005). Differential cytokine production and Toll-like receptor signaling pathways by *Candida albicans* blastoconidia and hyphae. *Infect. Immun.* 73 7458–7464 10.1128/IAI.73.11.7458-7464.2005 16239547 PMC1273874

[B19] Vonk A.G. Netea M. G. van Krieken J. H. Iwakura Y. van der Meer J. W. Kullberg B. J. (2006). Endogenous interleukin (IL)-1 alpha and IL-1 beta are crucial for host defense against disseminated candidiasis. *J. Infect. Dis.* 193 1419–1426 10.1086/503363 16619190

[B20] Zelante T. De Luca A. Bonifazi P. Montagnoli C. Bozza S. Moretti S. (2007). IL-23 and the Th17 pathway promote inflammation and impair antifungal immune resistance. *Eur. J. Immunol.* 37 2695–2706 10.1002/eji.200737409 17899546

